# Toward a new framework for the development of individualized therapies

**DOI:** 10.1038/s41434-020-0143-y

**Published:** 2020-04-02

**Authors:** Peter Marks, Celia Witten

**Affiliations:** Center for Biologics Evaluation and Research, US Food and Drug Administration, Silver Spring, MD USA

**Keywords:** Gene therapy, Diseases

The concept of personalized medicine—selecting the appropriate previously developed treatment off the shelf to address an individual’s condition—now has been around for decades. As the era of molecular medicine matures, however, it is becoming increasingly apparent that individualized therapeutics developed for one or a few individuals will become central to addressing the unique molecular defects present in hundreds to thousands of genetic or acquired diseases through the application of any one of several different technologies. The recent appreciation of the potential for bespoke therapeutics to address serious unmet medical needs has highlighted the need for a shift to an end-to-end approach enabling the provision of individualized therapeutic products, most notably in the areas of directly administered vectored gene therapy, genetically modified cellular therapies, and antisense oligonucleotides (ASOs) [1, 2]. Foundational to enabling an individualized approach to product development will be the development of a regulatory framework that supports such development in a way that is fundamentally different in certain respects from the approach that has been taken in the past for products aimed for the treatment of tens or hundreds of thousands of individuals.

As the framework is developed, it should be in the context of an appropriate paradigm that facilitates ethical and safe patient treatment under investigational new drug (IND) application regulations [3]. The features of this framework will need to be developed in a markedly accelerated manner in the coming months, ideally building upon existing statutory authorities, rather than requiring new ones, as it is increasingly clear that the development of such products for the treatment of one to up to a few hundred individuals will soon become routine, rather than an exception. Given the novelty and potential importance of this new framework to those in need of such treatments, FDA appreciates that a transparent process for its development will be important with input from patients, academics, industry, and other stakeholder groups.

In addition to developing an appropriate regulatory framework, other issues for these products include determining the quantity of supportive preclinical evidence needed prior to patient treatment, the production of quality product fit for purpose, and understanding the relevant disease-specific clinical information that should be captured when patients are treated. In addition, a sustainable way will be needed to deliver these products to individuals if they show promise, since they are currently not generally considered to be viable from a commercial perspective [4].

Focusing in on directly administered gene therapies using adeno-associated viral (AAV) vectors provides a case study that might be informative to other situations, such as that for certain genetically modified cellular therapies and for ASOs. In the case of AAV, current manufacturing constraints present a sort of goldilocks phenomenon, in which viable commercial solutions are not available for very small or large populations but do exist for mid-size indications (Fig. 1).

**Fig. 1:**
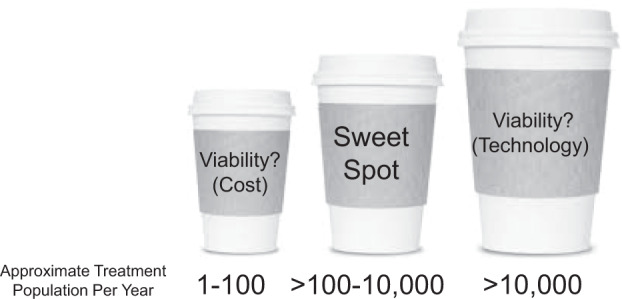
The current capacity for manufacturing of adeno-associated viral (AAV) gene therapy vectors is best suited for medium size populations ranging from roughly >100 to 10,000 individuals. Current constrains on the technology for production related to the existing expression systems for making AAV preclude the production of the necessary quantities of product that would be required to address larger patient populations. At the other end of the spectrum, the current costs associated with product development preclude commercial viability of products that would be used in a very small number of individuals each year.

Indeed, the complexities of scale-up and purification have been reasonably solved for gene therapies addressing moderate-sized populations of hundreds to several thousand individuals. However, current cell lines used for vector production are sufficiently inefficient, such that the production of a gene therapy for tens of thousands of individuals is not possible at this time, even though such products are conceptually possible. A hypothetical example of such a broadly applicable therapy might be an AAV-vectored antisense gene therapy or gene editing therapy that reduces proprotein convertase subtilisin/kexin type 9 levels by half [5]. This might be an approach for reduction in cardiovascular disease to those at increased risk following cardiac events, among other possible applications.

At the other end of the spectrum, many rare genetic disorders that are associated with significant morbidity and mortality affect very small populations ranging from one to a hundred individuals. The need in this area is perhaps more pressing than for the scale-up of gene therapies to large populations. This is because we currently have the potential to address many very rare conditions, but manufacturing high-quality AAV vectors for clinical trials to be conducted in such small populations can be cost prohibitive. In addition, such products, often developed in academic settings, are unattractive commercially, and are unlikely to find a commercial sponsor to facilitate their availability over the long term even if evidence indicates that they are safe and effective. The FDA is committed to proactively working with a range of federal, industrial, academic, and advocacy organizations to address the unmet medical need in this area and believes that a public–private partnership, potentially making use of a consortium model, might best address the critical need for AAV-vectored gene therapies and other individualized therapies.

The mission of a public–private partnership applied to AAV-vectored gene therapy would be to provide access to bespoke gene therapy products addressing serious unmet medical needs. The partnership could provide end-to-end solutions for key issues limiting the development and application of gene therapy to very rare genetic abnormalities, including advancing product clinical development through use of innovative Bayesian and adaptive trial designs and by facilitating product availability prior to and following any potential regulatory approval. It is anticipated that addressing the need for individualized gene therapy products will also lead to scientific, technical, and regulatory developments that could advance the entire field of gene therapy, particularly as it advances into the realm of genome editing.

It may be useful to provide a few more specific examples regarding how this approach might address existing bottlenecks in the gene therapy development process for very rare disorders. First, although academic investigators who interact with affected patients are often in the best position to help develop therapies for very rare disorders, they often lack experience with AAV manufacturing or advanced product development leading to regulatory filings. The production by a consortium of a non-proprietary suite of AAV vectors, along with detailed instructions for developing the construct that would address the genetic disorder, could increase the number of products in development addressing unmet medical needs. Second, the challenges in terms of cost and time associated with manufacturing small batches of material in accordance with current Good Manufacturing Practice in an academic facility or the lack of access to contract manufacturing organizations with the capacity to produce product in a timely manner at an affordable price could be addressed by having products manufactured at one or a few facilities according to standardized procedures that could be documented for regulatory purposes in master files, reducing the regulatory burden associated with the production of each new product. Third, the challenges that academic researchers unfamiliar with regulatory processes, such as submitting an IND application, often face could be at least partially addressed by the development of IND and protocol templates. Fourth, for those patients falling outside of the eligibility criteria of a protocol to receive a product, the consortium could assist with the development of uniform policies for expanded access to the products. Finally, the crucial issue of the continued production of such gene therapy products for patients requiring individualized therapies targeted to very small populations could be facilitated through the public–private consortium.

In addition, with appropriate careful scientific and technical planning, gene therapies that ultimately turn out to be useful in the treatment of somewhat larger populations, which have the potential to be commercially viable, could potentially be seamlessly transferred from nonproprietary to proprietary systems. Such product out-licensing, in combination with licensing of any advanced production technologies that are incidentally developed, could potentially help sustain a public–private partnership financially.

Most importantly, by addressing several of the issues for very rare product development that are not well addressed in the regulatory framework as it exists today, it is expected that the average time for the development of a therapy for a very rare disorder could be reduced by at least half (Fig. 2). This approach will potentially require a flexible approach by regulatory authorities, which are more used to dealing with conventional development programs. However, the time is ripe for addressing these challenges, as an increasing number of individualized products are entering the pipeline.

**Fig. 2:**
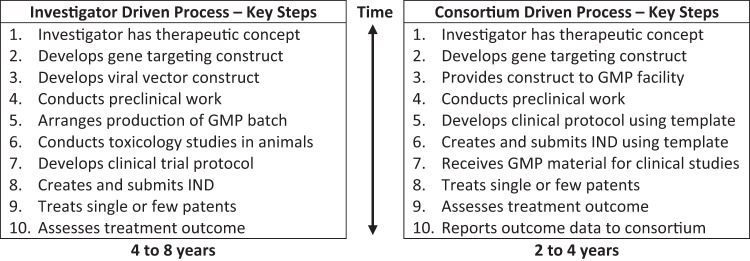
The potential benefits of a public–private consortium approach to the manufacturing of individualize gene therapy products include a significant reduction in the time for development. The gene therapy product that would be produced with high quality at a site with significant manufacturing experience. Through use of templates and reference to master regulatory filings, it is expected that the burden of regulatory submissions could be reduced, and that ultimately the time from conception of a treatment to assessment of its effect could be reduced by about half or more.

The successful development of an appropriate regulatory framework, combined with the accomplishment of a consortium initiative for AAV might also serve as paradigm for the development of other individualized therapies, such as genetically modified T cell therapies targeting multiple antigens unique to an individual’s cancer and for ASOs addressing individual or very rare mutations. For such a paradigm to be most successful for patients affected with very rare diseases globally, there should be international alignment between regulatory authorities, allowing for the use of individualized products developed and produced in one regulatory jurisdiction to be readily available to those in need in other jurisdictions.

Individualized, or bespoke, therapeutics are now forcing us to rethink our current manufacturing, preclinical and clinical development, and regulatory paradigms. This is a very welcome development if it ultimately results in the timely delivery of safe and effective medical products that transform patients’ lives.

## References

[1] Prickett TD, Crystal JS, Cohen CJ, Shamalov K, Trebska-McGowan K, Bliskovsky VV (2016). Durable complete response from metastatic melanoma after transfer of autologous T cells recognizing 10 mutated tumor antigens. Cancer Immunol Res.

[2] Kim J, Hu CA, Moufawad El Achkar C, Black LE, Douville J, Larson A (2019). Patient-customized oligonucleotide therapy for a rare genetic disease. N Engl J Med.

[3] Woodcock J, Marks P (2019). Drug regulation in the era of individualized therapies. N Engl J Med.

[4] Hampson G, Towse A, Pearson SD, Dreitlein WB, Henshall C (2018). Gene therapy: evidence, value and affordability in the US health care system. J Comp Eff Res.

[5] King A (2018). A CRISPR edit for heart disease. Nature.

